# Editorial: Cartilage assessment using quantitative MRI

**DOI:** 10.3389/fendo.2022.1092354

**Published:** 2022-11-29

**Authors:** Saeed Jerban, Edwin H. G. Oei, Jianping Ding

**Affiliations:** ^1^ Department of Radiology, University of California, San Diego, San Diego, CA, United States; ^2^ Department of Radiology & Nuclear Medicine, Erasmus Medical Center (MC), University Medical Center, Rotterdam, Netherlands; ^3^ Department of Radiology, Affiliated Hospital of Hangzhou Normal University, Hangzhou, China

**Keywords:** magnetic resonance imaging, cartilage, osteoarthritis, knee joint, quantitative imaging

Cartilage degeneration coupled with aging or with degenerative diseases such as osteoarthritis (OA) affects a large number of global citizens (1). It is essential to access accurate non-invasive imaging techniques to characterize cartilage quality and health. This would allow earlier diagnosis and initiation of therapy, which is more effective at an early stage compared with a later stage when irreversible damage has occurred (2). These techniques are also crucial for follow-up evaluations after therapy. Magnetic resonance imaging (MRI) is considered the most sensitive and specific imaging tool for cartilage assessment. Quantitative MRI is capable of non-invasive assessment of cartilage at high spatial resolution and allows assessment of longitudinal changes in cartilage. This Research Topic is dedicated to cartilage assessment using quantitative MRI.

Quantitative MRI performed on cartilage can be classified into three following domains. A) quantitative morphometric MRI analysis, such as tibiofemoral cartilage thickness assessment can be performed using conventional 3D MRI sequences to investigate the thickness or volume changes over time in patients with OA (3). B) semiquantitative MRI grading techniques for cartilage assessment based on the morphological changes and the lesion appearance in the joint. For example, the whole-organ MRI score (WORMS) (4) and the MRI osteoarthritis knee score (MOAKS) (5) are examples of the scoring system used for the past two decades in several OA-related studies worldwide. C) quantitative MRI techniques that have been developed to characterize the tissue matrix quality and composition. These techniques have been also described before as compositional MRI and are hypothesized to be sensitive to glycosaminoglycan (GAG), water, and collagen changes in cartilage. T2 (transverse plane magnetic relaxation, spin-spin), T1 (longitudinal plane magnetic relaxation, spin-lattice), T1ρ (spin-lattice relaxation in the rotating frame), delayed gadolinium-enhanced MRI (dGEMRIC), and glycosaminoglycan chemical exchange saturation transfer imaging (GAG-CEST), diffusion MR imaging are examples of quantitative MRI techniques (6).

In this Research Topic, Chen et al. evaluated different quantitative MRI-based measures such as cartilage volume, thickness, and T2 relaxation time in meniscus tear patients in comparison to healthy volunteers. The articular cartilage T2 values in all subregions of the femur and tibia in the meniscus tear group were found significantly higher than in the healthy control group. T2 mapping potentially enables the detection of cartilage degeneration caused by meniscal tears. Liu et al compared the effects of long walking and regular daily physical training on the T2 values in cartilage and knee joint injuries. Both long-walking and daily physical training groups showed reduced T2 values of the knee joint compared to baseline. Knee cartilage thickness decreased in the long-walking group. The prevalence of medial meniscus and anterior cruciate ligament injuries of the knee joint, joint effusion, and bone marrow edema was significantly higher in the long-walking group compared to the baseline and daily physical training groups.

Short T2 values in the deep layer and the calcified zones of articular cartilage make imaging and quantifications challenging when conventional quantitative MRI techniques are used. Such tissues demonstrate little or no signal when scanned with conventional clinical sequences, which typically utilize TEs of several milliseconds. Ultrashort echo time (UTE) sequences with TEs 100-1000 times shorter than those of conventional clinical sequences can directly image short-T2 tissues in the knee joint, particularly the meniscus and the deep layer of the articular cartilage (7). In this Research Topic, Afsahi et al reviewed the articular cartilage assessment using UTE MRI techniques, including UTE T1, T2, T2* (apparent transverse plane relaxation), T1ρ, magnetization transfer (MT), double echo steady state (DESS), quantitative susceptibility mapping (QSM) and inversion recovery (IR). A major confounding factor in several quantitative MRI techniques, such as T2 and T1ρ, is the magic angle effect, which may result in a several-fold increase in MR quantifications depending on the tissue fibers’ orientation inside the scanner. Among the described quantitative UTE-MRI techniques, UTE Adiabatic T1ρ and UTE-MT modeling techniques have demonstrated low sensitivity levels to the magic angle effect.

Several studies in the literature have focused on quantitative MRI for the evaluation of the osteochondral junction (OCJ), where the cartilage-bone transition occurs and the T2 of the tissue is very short (8, 9). The presence of fat in bone marrow located near OCJ can introduce more challenges to its imaging and quantification. In this Research Topic, Jang et al presented the feasibility of using a combination of inversion recovery and fat saturation preparation pulses with the zero echo time acquisition (IR-FS-ZTE) technique for osteochondral junction imaging. In an ex vivo experiment, IR-FS-ZTE produced improved imaging of the OCJ region over the clinical sequences, and significantly improved the contrast compared to FS-ZTE without IR preparation.

The guest editors thank all the authors for their outstanding contributions to this Research Topic. We hope the readers will be as excited as we are to apply quantitative MRI biomarkers for cartilage assessment in clinical studies.

## Author contributions

All authors listed have made a substantial, direct, and intellectual contribution to the work and approved it for publication.

## Funding

The authors acknowledge grant support from the National Institute of Arthritis and Musculoskeletal and Skin Diseases (NIAMS) (K01AR080257).

## Conflict of interest

The authors declare that the research was conducted in the absence of any commercial or financial relationships that could be construed as a potential conflict of interest.

## Publisher’s note

All claims expressed in this article are solely those of the authors and do not necessarily represent those of their affiliated organizations, or those of the publisher, the editors and the reviewers. Any product that may be evaluated in this article, or claim that may be made by its manufacturer, is not guaranteed or endorsed by the publisher.
